# Are Older Patients with Cervical Cancer Managed Differently to Younger Patients? An International Survey

**DOI:** 10.3390/cancers11121955

**Published:** 2019-12-06

**Authors:** Maxime Frelaut, Nienke De Glas, Ignacio Zapardiel, Orit Kaidar-Person, Maria Kfoury, Benoit You, Susana Banerjee, Etienne Brain, Claire Falandry, Manuel Rodrigues

**Affiliations:** 1 Department of Medical Oncology, Institut Curie, Paris Science & Lettres Research University, 75005 Paris, France; maxime.frelaut@curie.fr (M.F.); etienne.brain@curie.fr (E.B.); 2 Internal Medicine, Leiden University Medical Center, 2316 Leiden, The Netherlands; N.A.de_Glas@lumc.nl; 3 Gynecologic Oncology Unit, La Paz University Hospital-IdiPAZ, 28046 Madrid, Spain; ignaciozapardiel@hotmail.com; 4 Division of Oncology, Radiotherapy Unit, Rambam Health Care Campus, 31096 Haifa, Israel; o_person@rambam.health.gov.il; 5 Association d’Enseignement de Recherche des Internes en Oncologie, 75005 Paris, France; kfoury.maria@gmail.com; 6 Department of Medical Oncology, Lyon Sud Hospital Center, Centre d’Investigation des Thérapeutiques en Oncologie et Hématologie de Lyon (CITOHL), Institute of Cancerology, Hospices Civils de Lyon (IC-HCL), 69002 Lyon, France; benoit.you@chu-lyon.fr; 7 Unité Ciblage Thérapeutique en Oncologie Université Claude Bernard Lyon 1, Hospices Civils de Lyon 3738, Faculty of Medicine-Lyon Sud, University of Lyon 1, 69600 Oullins, France; 8 Gynae Oncology Unit, The Royal Marsden NHS Foundation Trust, London SW3 6JJ, UK; Susana.Banerjee@rmh.nhs.uk; 9 Geriatric Unit, Centre Hospitalier Lyon Sud, 69 495 Pierre-Bénite, France; claire.falandry@chu-lyon.fr; 10 CarMen biomedical research laboratory (Cardiovascular diseases, Metabolism, diabetology and Nutrition) Institut national de la santé et de la recherche médicale (INSERM) U1060, Université de Lyon, 69600 Oullins, France; 11 Institut Curie, Institut national de la santé et de la recherche médicale (INSERM), PSL Research University, U830, 75005 Paris, France

**Keywords:** cervical cancer, oncogeriatrics, radiotherapy, chemotherapy, surgery

## Abstract

Although a quarter of cervical cancers occur after the age of 65 years, there is no treatment consensus for these patients. The aim of this work was to survey how physicians treat patients with advanced cervical cancer, focusing on treatment adjustments according to age and frailty status. Specialists were invited to an online survey. Data collected included information on respondent and treatment strategy in four cases (FIGO IIb, FIGO IVa, FIGO IVb, metastatic recurrence) with three age scenarios (45-year-old, 75-year-old and fit, 75-year-old and unfit). We received 237 responses of which 117 were fully completed. Thirty-four percent of respondents reported they had available access to a geriatric team and 25% used a frailty screening tool in routine. Therapeutic strategies did not differ between young and old fit patients. However, treatment modalities and intensity were different for old and unfit patients. Physicians answered that they would treat old fit patients as their younger counterparts but would reduce treatment intensity for old unfit patients. However, even if they were willing to adapt their treatment strategy based on frailty status, most of them do not use the tools that would allow distinguishing “fit” and “unfit” older patients, leaving room for improving accurate geriatric evaluation.

## 1. Introduction

Cervical cancer is the second most common cancer in women with 500.000 new cases every year. While the global incidence has decreased with the implementation of screening programs, and may be improved by the use of tumor markers [1], the incidence in older women has remained unchanged, with a quarter of cases occurring after the age of 65 years [2]. Moreover, older patients often present with later stages of cervical cancers [3]. Treatment of localized cervical cancer includes surgery, brachytherapy, and concomitant radio-chemotherapy [4]. However, the benefit-risk balance for these treatments among older patients has been poorly studied. Treatment decisions in older patients are complex due to frequent comorbidities and age-related impairments, such as malnutrition, functional dependence, and cognitive decline [5]. Moreover, older patients are at increased risk of treatment toxicity [6]. For all these reasons, treatment of cervical carcinoma is not consistent in the geriatric population. The aim of this study was to survey the treatment attitudes of gynecological cancer specialists for cervical cancer according to age and health status.

## 2. Materials and Methods 

A survey designed by a core group of international multidisciplinary specialists according to Burns et al. was sent to physicians around the world who treat patients with cervical cancer [7]. The survey was created with the LimeSurvey online tool, and was approved by the institutional review board of the Institut Curie, Paris, France (registration number: DATA190199). Participation was voluntary and responses were anonymous. The questionnaire was anonymously sent by email to gynecological surgeons, radiotherapists, medical oncologists, and geriatricians through networks from the young committee of the Société Internationale d’Oncologie Gériatrique (Young SIOG), the young network of the European Society of Gynaecological Oncology (ENYGO), the European Organization for Research, and Treatment of Cancer (EORTC; gynaecological cancer group, radiation oncology group, and cancer in elderly task force), and the *Association d’Enseignement et de Recherche des Internes en Oncologie* (AERIO, French national association of residents in oncology). Since the exact number of recipients and duplicates (people present in at least two mailing lists) were unknown, the response rate could not be estimated.

The survey was divided in two parts (supplementary data, Online Survey). In the first part, general professional characteristics of respondents were collected, including description of practice and local context. In the second part, respondents were asked to give their treatment choices for cervical cancer in twelve clinical scenarios. These clinical cases related to three patients presenting cervical cancers at four different stages. The three patients were (a) a 45-year-old (yo) woman with no significant comorbidity (young, fit), (b) a fully independent 75 yo woman with no comorbidity and with available caregivers (old, fit), (c) an isolated 75 yo woman with no severe comorbidity but dependent for shopping, cleaning, and went out only twice a week (old, unfit). The four different stages were (i) a 50 mm FIGO IIb cervix cancer with bilateral proximal parametrial invasion, (ii) a 50 mm FIGO IVa cervix cancer with bilateral proximal parametrial invasion and rectal wall invasion, (iii) a 50 mm FIGO IVb cancer with lung and liver asymptomatic metastases, and (iv) an asymptomatic lung and liver metastatic recurrence without local recurrence five years after chemo-radiation. Standard treatment procedures were defined for each case according to the European Society of Medical Oncology guidelines [4]. Only completed questionnaires were included in the analysis. 

Statistical analyses were performed using the R Statistical Software (version 3.2.5; R Foundation for Statistical Computing, Vienna, Austria). We compared the therapeutic proposals in each case between (i) the old fit and young fit patients and (ii) between old unfit and young fit patients. Since these analyses were performed with the aim to assess differences in the proposals were made, we chose to use only descriptive statistical methods without additional adjustments using the Fisher’s *p* exact test with an alpha risk of 5%. 

## 3. Results

Two hundred thirty-seven replies from 26 countries were received from June to November 2015. Among them, 117 questionnaires were fully completed (49%) and were included in the analysis. The median age of respondents was 38 yo (range 27–76; Table 1). Fifty-five were surgeons (47%), 33 (28%) were radiation oncologists, 52 (44%) were chemotherapy prescribers (among them 14 were surgeons and 18 radiation oncologists), and 16 (14%) were geriatricians or physicians trained in geriatric oncology. Most respondents practiced in public institutions (*n* = 93; 79%), and 51 (44%) supervised junior doctors. The respondents declared treating a median of 25 new patients with cervical cancer every year, including five older than 70 yo. Half of respondents (*n* = 59) treated other cancer types (breast cancer, 38%; genito-urinary cancers, 27%; digestive cancers, 21%; sarcomas, 18%; thoracic cancers, 14%). The majority had access to radiotherapy (*n* = 108; 92%), brachytherapy (*n* = 98; 84%), and chemotherapy (*n* = 104; 89%). Seventy-seven (66%) had access to bevacizumab in the metastatic setting. Of the 101 non-geriatrician respondents, 40 (34%) could refer patients to a geriatric team to perform a comprehensive geriatric assessment when needed. Only 29 of all respondents (25%) and 16 (40%) of respondents with access to a geriatric team declared using routinely a frailty screening tool. The most frequently used tools were the G8 (*n* = 15/29; 52%), the VES-13 (*n* = 8/29; 26%), and the GFI (*n* = 5/29; 17%). 

The treatment strategies they reported for the clinical cases are reported in Table 2. For stage IIB disease, standard treatment (i.e., concomitant radiochemotherapy) was proposed by 59%, 64%, and 47% of respondents in the 45 yo, 75 yo fit (*p* = 0.33), and 75 yo unfit patient (*p* = 0.03), respectively. For the old unfit patient, the main alternative to standard treatment consisted in radiotherapy alone. For stage IVA disease, standard treatment (i.e., concomitant radiochemotherapy) was proposed by 62%, 59%, and 35% of respondents for the 45 yo, 75 yo fit (*p* = 0.71), and 75 yo unfit patient (*p* < 0.001), respectively. For the old unfit patient, the main alternative to standard treatment consisted again in radiotherapy alone. For stage IVB disease, standard chemotherapy doublet with platinum was proposed by 60%, 55%, and 36% of for the 45 yo, 75 yo fit patient (*p* = 0.51), and 75 yo unfit patient (*p* = 0.001), respectively. Physicians said they would add bevacizumab to chemotherapy in 42% of cases for the 45 yo woman, 33% for the 75 yo fit woman (*p* = 0.22) and 12% (*p* < 0.001) for the 75 yo unfit patient. Supportive care alone was the main alternative to chemotherapy doublet for the 75 yo unfit patient. For recurrent disease, 83%, 78%, and 54% of respondents proposed chemotherapy to the 45 yo, 75 yo fit patient (*p* = 0.28) and 75 yo unfit patient (*p* < 0.001), respectively. Among them 65%, 57%, and 33%, respectively, answer they would add bevacizumab to chemotherapy. Supportive care alone was the main alternative to chemotherapy for the 75 yo unfit patient.

## 4. Discussion

In this survey, physicians answered that they would treat old fit patients as their younger counterparts, but would change their therapeutic proposition to less intensive treatments for old, unfit patients in all clinical scenarios. However, of the 117 cancer specialists who responded to this survey, only 25% use frailty screening tools, and one third has access to a geriatric team. This work suggests that a high level of subjectivity persists for treatment selection as physicians want to personalize their treatment recommendations according to frailty assessment, but do not have access to appropriate assessments that would allow them to do so. 

In a previous study, Gao et al. retrospectively collected data from 159 patients with locally advanced cervical cancer treated in one center in China between January 2007 and January 2009, dividing their cohort between patients ≥ 65 years-old and < 65 years-old [8]. Among older patients, the most common treatment was radiotherapy alone, while the more frequent treatment among younger patients was radiochemotherapy. More recently, De Boer et al. developed a survey to assess patterns of care in cervical cancer, but they focused only on radiotherapy modalities, and in a single country [9]. As in our work, Hamamoto et al. observed in a Japanese study on esophageal carcinoma that the preferred strategy in fit older patients was the same as in younger ones [10]. Once again, similarly to our study, radiation alone was preferred in frail patients. To our knowledge, our work is the first international study to survey clinical practices of gynecologic oncology physicians for older patients with cervical cancer. However, our survey has several limitations. First, it suffers generic intrinsic limitations such as a bias of recruitment in favor of responders with a higher interest for geriatric oncology than the overall population of physicians. To circumvent this bias, we not only solicited geriatric oncologists through SIOG, but also medical oncology, surgery, and radiotherapy groups. Another recruitment bias resulted in a relative young age of responders (median of 38 yo). This is likely to be explained because the survey was relayed by two groups of young physicians (ENYGO and AERIO), resulting in less experienced responders. These younger physicians certainly have less experience than older ones, however, we believe that they are sufficiently experienced to know international guidelines and apply them, whenever possible. However, in our study, only ~60–80% of physicians proposed a standard treatment according to international recommendations. This low rate is explained by our very stringent interpretation of answers; i.e., if the response did not correspond perfectly to the standard of care, it was interpreted as “non-standard”. Furthermore, local practice and inequitable access to medical resources between respondents may also have result in discrepancies between respondents. Such discrepancies have been reported in a large EORTC survey [11]. Oosting et al. reported, for instance, that one third of investigated centers do not treat older patients suffering with oropharyngeal carcinoma with chemoradiation, while 16% of centers treat more than 40% of these patients with such modality. Similarly, more than half of the centers never use cetuximab in older patients with hypopharyngeal carcinoma, while 20% treat more than 20% of these patients with this antibody. In our study, we circumvented these issues by observing how physicians adapt their treatment proposals to different situations. Respondents were their own controls, thus compensating the inequalities and discrepancies between centers and countries. 

There is no established evidence that treatment in the second-line setting for cervical cancer improves overall survival compared with best supportive care. Treatment options that offer improvement in disease-related symptoms, quality of life, and prolongation of progression-free survival are worthwhile [12]. Notably, immune checkpoint programmed cell death 1 (PD-1) and T-lymphocyte–associated molecule-4 (CTLA-4) inhibition may represent a robust strategy to overcome immune suppression and improved outcome, with ongoing phase III studies [13]. However, decreased immunity due to aging may be a limitation for such therapeutic approach in the elderly patients. Besides, treatment of advanced cervical cancers in older patients remains controversial and poorly defined. A previous analysis of the American Surveillance, Epidemiology, and End Results program showed that older women with cervical cancer are treated differently compared with their younger counterparts, with less primary surgery for early-stage (33% versus 55% versus 82% in those >80 yo, >70 yo, and <50 yo, respectively) [14]. Similarly, a recent review by Venkatesulu et al. suggests that older patients receive less intensive treatments because of anticipated morbidity [15]. To which extent this decision could contribute to the decreased survival observed in older patients (85% five-year overall survival for ≤45 yo versus 29% for ≥75 yo patients) remains a question mark [16]. In our study, respondents proposed the same treatment strategy for younger adults and fit older patients, restricting adjustments to those who were unfit. However, because of the lack of studies in this population, it is unclear if we should treat older fit patients exactly as younger ones. For instance, radiochemotherapy with concomitant cisplatin has been the standard of care for localized advanced cervix cancer for the last 20 years, but its benefit in older is unclear since no patient over 65yo was included in radiochemotherapy randomized clinical trials [17]. In a retrospective study with 105 patients over 65yo, Park et al. did not observe survival benefit with the addition of chemotherapy to radiotherapy for stage IB2 to IVA cervical cancer in patients over 65 yo, but a clear increase in hematological toxicities [18]. Older patients are more exposed to adverse events, such as renal failure, vomiting, proctitis, cystitis, or denutrition, reflecting frailty. However, other retrospective studies claimed that radiochemotherapy is well tolerated and effective in these older populations, but with wrong definition of ‘elderly’ (i.e., over 60 or 65 years old) [19,20,21]. Eventually, none of these studies used scales to assess specific outcomes in this geriatric population, precluding solid recommendations regarding radiochemotherapy on older patients. This clearly underlines the need for more specific prospective works to evaluate the efficacy and tolerance of these treatments in older patients.

Despite strong demonstration, reducing treatment intensity in unfit patients remains a rational, pragmatic and careful decision. Noteworthy, respondents reported frequent reduction of treatment intensity in case of unfit status, but the standard treatment was still the preferred option. This observation questions current clinical practices for older patients with cancer, as highlighted by the rare use of a screening tool for frailty and the low referral to dedicated multidisciplinary teams. Similarly, Hamamoto et al. observed that geriatric scales were not a main factor for decision-making in this population [10], while Oosting et al. reported that only 13% of centers routinely perform geriatric assessment in their international survey [11]. All older patients, from age 70–75, with cancer should be screened for frailty to streamline resource and time [5]. Those classified at a high risk of frailty should then be evaluated by a geriatric multidisciplinary team before treatment initiation. Applying these recommendations could help physicians to adapt the treatment of older patients with cervical cancer to their functional status and not their chronological age [22,23]. Several explanations may be considered for the little use of frailty screening tools. Some respondents may think that these tools are not useful. Screening tools present high sensitivity, thus helping physicians in their daily practice to identify high-risk patients [24]. Another possible explanation is that respondents may think that they do not have enough time to these tools in consultation. However, any caregiver can easily use screening tools and studies have shown that they can be filled in five minutes or less. Use of screening tools is strongly recommended, they should be widely disseminated, in order to streamline the access to comprehensive geriatric assessment. Our study highlights the need for improvement in geriatric oncology practices among all health professionals, whatever their age or experience. Health policies should facilitate access to geriatrics and support training in geriatric oncology. 

## 5. Conclusions

In our survey, specialists declared that they would treat old fit patients as younger patients with advanced or metastatic cervical cancers, while they proposed less intensive treatments for old unfit patients. However, they were a large majority to not use an appropriate geriatric assessment in their current practice to identify these patients, such as frailty screening scales and multidisciplinary geriatric evaluation. This work emphasizes the need to promote training in geriatric oncology and subsequent use of frailty screening scales in daily practice.

## Figures and Tables

**Table 1:** Characteristics of respondents.

Characteristics	Median (Range)
Median age	38(27–76)
Median number of new patients > 70 yo with cervix cancer seen yearly	5 (0–50)
**Characteristics**	**% (numbers)**
Practice	
Surgery	47 (55)
Radiotherapy	28 (33)
Chemotherapy	44 (52)
Type of practice	
Public	79 (93)
Private	17 (20)
Junior doctors supervisors	44 (51)
Treating other cancers	
Yes	50 (59)
Breast cancer	38 (44)
Genito-urinary cancers	27 (32)
Digestive cancers	21 (24)
Sarcomas	18 (21)
Thoracic cancer	14 (16)
Melanoma	9 (10)
Cerebral tumors	9 (11)
Head and neck cancers	9 (11)
Treatment access	
Radiotherapy	92 (108)
Intensity-modulated radiotherapy	65 (75)
Brachytherapy	84 (98)
Low pulse-dose rate brachytherapy	45 (38)
Chemotherapy	89 (104)
Bevacizumab	66 (77)

**Table 2:** Treatment propositions for each case and patient.

Treatment	45 yo Patient % (Nb)	75 yo Fit Patient % (Nb; *p* *)	75 yo Unfit Patient % (Nb; *p* **)
**Case i (IIb)**			
Radiochemotherapy +/− surgery	59 (69)	64 (75; *p* = 0.33)	**47 (55; *p* = 0.03)**
Radiotherapy alone	2 (2)	2 (2; *p* = 1)	**20 (23, *p* < 0.001)**
Chemotherapy then radiochemotherapy	7 (8)	6 (7, *p* = 1)	4 (5, *p* = 0.57)
Chemotherapy then surgery	8 (9)	3 (4, *p* = 0.25)	3 (4, *p* = 0.25)
Surgery alone	5 (6)	3 (4; *p* = 0.75)	3 (4; *p* = 0.75)
Other	19 (23)	22 (25; *p* = 0.87)	22 (26; *p* = 0.75)
**Case ii (IVa)**			
Radiochemotherapy +/− surgery	62 (72)	59 (69; *p* = 0.71)	**35 (41; *p* < 0.001)**
Radiotherapy alone	2 (2)	1 (1; *p* = 1)	**20 (23; *p* < 0.001)**
Chemotherapy then radiochemotherapy	17 (20)	15 (17; *p* = 0.72)	10 (12; *p* = 0.13)
Chemotherapy then surgery	3 (4)	1 (1; *p* = 0.37)	3 (3; *p* = 0.72)
Supportive care only	0	0	**6 (7; *p* = 0.01)**
Other	16 (17)	25 (27; *p* = 0.13)	**26 (31; *p* = 0.03)**
**Case iii (IVb)**			
Chemotherapy doublet with platinum	60 (70)	55 (64; *p* = 0.51)	**36 (42; *p* = 0.001)**
Other chemotherapy +/− radiochemotherapy	12 (15)	15 (19; *p* = 0.58)	23 (26; *p* = 0.08)
Bevacizumab	42 (49)	33 (39; *p* = 0.22)	**12 (15; *p* < 0.001)**
Radiochemotherapy	10 (12)	10 (12; *p* = 1)	11 (13; *p* = 1)
Supportive care only	1 (1)	4 (5; *p* = 0.21)	**15 (17; *p* < 0.001)**
Other	16 (19)	15 (17; *p* = 0.86)	16 (19; *p* = 1)
**Case iv (metastatic recurrence)**			
Chemotherapy	83 (97)	78 (91; *p* = 0.28)	**54 (63; *p* < 0.001)**
Bevacizumab	54 (63)	44 (52; *p* = 0.19)	**18 (21; *p* < 0.001)**
Chemotherapy if symptomatic	8 (9)	11 (13; *p* = 0.5)	**18 (21; *p* = 0.001)**
Supportive care only	0	2 (2; *p* = 0.5)	**19 (22; *p* < 0.001)**
Others	9 (11)	9 (11; *p* = 1)	9 (11; *p* = 1)

*p* < 0.05 are in bold. yo: year-old; * Comparison between 75 year-old fit patient and 45 year-old patient; ** Comparison between 75 year-old unfit patient and 45 year-old patient.

## Supplementary Materials

The following are available online at https://www.mdpi.com/2072-6694/11/12/1955/s1, Online Survey.
