# Supplementary material for: Are Older Patients with Cervical Cancer Managed Differently to Younger Patients? An International Survey
Source: Cancers (Basel). 2019 Dec 6;11(12):1955. doi: 10.3390/cancers11121955 (PMC6966543; doi:10.3390/cancers11121955)
Supplement: Supplementary file 1 [file cancers-11-01955-s001.pdf]

Cervix cancer care according to the age

This survey aims at studying the cervix cancer care of elderly patients by observing the current practices.

The survey is anonymous and should take you only few minutes.

There is no compulsory question, you can pass if you do not know an answer.

Thank you

There are 142 questions in this survey

General informations

[]How old are you?

Only an integer value may be entered in this field.

Please write your answer here:

[]What is your main practice type ?

Please choose **all** that apply:

- ☐ Public or equivalent
- ☐ Private
- ☐ I supervise doctors in training
- ☐ I don't supervise doctors in training

[]What are your professional practices?

Please choose **all** that apply:

- ☐ Chemotherapy
- ☐ Oncologic surgery
- ☐ Radiotherapy

[]In which country do you exercise?

Please choose **only one** of the following:

- Albania
- Andorra
- Armenia
- Austria
- Aserbaidschan
- Belgium
- Bosnia-Herzegovina
- Bulgaria
- Byelorussia
- Canada
- Croatia
- Cyprus
- Czech Republic
- Denmark
- Estonia
- Finland
- France
- Georgia
- Germany
- Greece
- Hungary
- Iceland
- Ireland
- Italy
- Kazakhstan
- Kosovo
- Latvia
- Liechtenstein
- Lithuania
- Luxemburg
- Macedonia
- Malta
- Moldavia
- Monaco
- Montenegro
- Netherlands
- Norway

- Poland
- Portugal
- Rumania
- Russia
- San Marino
- Serbia
- Slovakia
- Slovenia
- Spain
- Sweden
- Switzerland
- Turkey
- Ukraine
- United Kingdom
- USA
- Vatican
- Other

[]How many new patients with cervix cancer do you treat each year (estimation)?

Please write your answer(s) here:

New patients with cervix cancer

Among which new patients with cervix cancer older than 70 years

[]Do you treat other cancers than pelvic gynecological cancers?

Please choose **only one** of the following:

- Yes
- No

[]If yes

Only answer this question if the following conditions are met:
Answer was 'Yes' at question '6 [autrescancers]' (Do you treat other cancers than pelvic gynecological cancers?)

Please choose **all** that apply:

- ☐ Breast
- ☐ Thorax
- ☐ Genito-urinary
- ☐ Digestive
- ☐ Sarcoma
- ☐ Melanoma
- ☐ Brain
- ☐ Head & Neck
- ☐ Other: ______

[]For the treatment of cervix cancer you have access to:

Please choose **all** that apply:

- ☐ Radiotherapy
- ☐ Brachytherapy
- ☐ Chemotherapy (cisplatin, carboplatin, paclitaxel, 5-fluorouracil …)
- ☐ Bevacizumab in metastatic or relapsing cervix cancer

[]

In what type of radiotherapy have you access? *Clarify if you know*

Only answer this question if the following conditions are met:
Answer was at question '8 [dispo]' (For the treatment of cervix cancer you have access to:)

Please choose **all** that apply:

- ☐ Conventional
- ☐ IMRT

[]

In what type of brachytherapy have you access? *Clarify if you know*

Only answer this question if the following conditions are met:
Answer was at question '8 [dispo]' (For the treatment of cervix cancer you have access to:)

Please choose **all** that apply:

- ☐ Pulsed low dose rate
- ☐ High dose rate

[]Are you a specialist in oncogeriatrics?

Please choose **only one** of the following:

- ○ Yes
- ○ No

[]Do you use a frailties screening tool for elderly patients, and if yes which one(s)?

Please choose **all** that apply:

- ☐ No
- ☐ Oncodage G8
- ☐ Flemish Tirage Risk Screening Tool (TRST)
- ☐ Vulnerable Elders Survey-13 (VES-13)
- ☐ Gronningen Frailty Indicator (GFI)
- ☐ Other: ____________

[]Can you refer patients to a geriatric team for a comprehensive geriatric assessment (CGA)?

Please choose **only one** of the following:

- ○ Yes
- ○ No

The CGA (Comprehensive Geriatric Assessment) is a global evaluation of nutritional state, neuro-psychological state, autonomy, comorbidities and social conditions using specific questionnaires during a multi-disciplinary interview with dietitian, social worker, geriatrician …

Clinical cases

[]

Here are 4 clinical cases of cervical cancers

All therapeutic options are presented in the same way in every cases.

Every cases will be discussed :

- for a 45 years old woman without comorbidity,

- for a 75 years old woman, without comorbidity, with a good performance status and a present entourage:

- For a 75 years old woman without severe comorbidity but with a limited autonomy.

Case 1 a

What is your current treatment for this type of cervix squamous carcinoma without lombo-aortic or pelvic involvement at the imaging?

Age (years)	45
FIGO	IIB
Size (mm)	50
Parametrial invasion	Bilateral proximal parametrial invasion
Adjacent organs invasion	No
Lymphovascular invasion	No
Visceral metastases	No

[]Do you perform a lombo-aortic surgical exploration?

Please choose **only one** of the following:

- Yes
- No

[]In the absence of lombo-aortic involvement, what is your treatment?

Please choose **only one** of the following:

- Surgery
- Radiotherapy or concomitant radio-chemotherapy and/or brachytherapy
- Radiotherapy or concomitant radio-chemotherapy and/or brachytherapy then surgery
- Chemotherapy then surgery
- Chemotherapy then radiotherapy or concomitant radio-chemotherapy and/or brachytherapy
- Chemotherapy alone
- Supportive care without specific cancer treatment

[]What chemotherapy regimen do you use?

Only answer this question if the following conditions are met:
Answer was 'Chemotherapy then surgery ' *or* 'Chemotherapy then radiotherapy or concomitant radio-chemotherapy and/or brachytherapy ' *or* 'Chemotherapy alone' at question '16 [ttt]' (In the absence of lombo-aortic involvement, what is your treatment?)

Please choose **only one** of the following:

- Cisplatin doublet
- Carboplatin doublet
- Cisplatin alone
- Carboplatin alone
- Other combination of cytotoxics
- Other

[]Do you add Bevacizumab?

Only answer this question if the following conditions are met:
Answer was 'Chemotherapy then surgery ' *or* 'Chemotherapy then radiotherapy or concomitant radio-chemotherapy and/or brachytherapy ' *or* 'Chemotherapy alone' at question '16 [ttt]' (In the absence of lombo-aortic involvement, what is your treatment?)

Please choose **only one** of the following:

- Yes
- No

[]What drug do you use in association with cisplatin?

Only answer this question if the following conditions are met:
Answer was 'Cisplatin doublet ' at question '17 [chimio]' (What chemotherapy regimen do you use?)

Please choose **only one** of the following:

- Paclitaxel
- Gemcitabine
- 5-fluorouracil
- Topotecan
- Don’t know
- Other

[]What drug do you use in association with carboplatin?

Only answer this question if the following conditions are met:
Answer was 'Carboplatin doublet' at question '17 [chimio]' (What chemotherapy regimen do you use?)

Please choose **only one** of the following:

- Paclitaxel
- Gemcitabine
- 5-fluorouracil
- Topotecan
- Don’t know
- Other

[]What drugs do you use?

Only answer this question if the following conditions are met:
Answer was 'Other combination of cytotoxics' at question '17 [chimio]' (What chemotherapy regimen do you use?)

Please write your answer(s) here:

First cytotoxic

Second cytotoxic

Third cytotoxic

[]Do you perform a pelvic external radiotherapy?

Only answer this question if the following conditions are met:
Answer was 'Chemotherapy then radiotherapy or concomitant radio-chemotherapy and/or brachytherapy ' *or* 'Radiotherapy or concomitant radio-chemotherapy and/or brachytherapy then surgery' *or* 'Radiotherapy or concomitant radio-chemotherapy and/or brachytherapy' at question '16 [ttt]' (In the absence of lombo-aortic involvement, what is your treatment?)

Please choose **only one** of the following:

- Yes
- No

[]What type of pelvic external radiotherapy do you perform?

Only answer this question if the following conditions are met:
Answer was 'Yes' at question '22 [rtexterne]' (Do you perform a pelvic external radiotherapy?)

Please choose **only one** of the following:

- Uterus, external, internal and primitive iliac nodes (up limit L4-L5): 45 Gy daily doses of 1.8-2.0 Grays
- Other regimen with adaptation of fields or physical parameters to reduce toxicity risks

[]Do you add concomitant chemotherapy to radiotherapy?

Only answer this question if the following conditions are met:
Answer was 'Yes' at question '22 [rtexterne]' (Do you perform a pelvic external radiotherapy?)

Please choose **only one** of the following:

- No
- Cisplatin alone
- Carboplatin alone
- Cisplatin+ 5-fluorouracil
- Other

[]Do you perform brachytherapy?

Only answer this question if the following conditions are met:
Answer was 'Radiotherapy or concomitant radio-chemotherapy and/or brachytherapy' *or* 'Radiotherapy or concomitant radio-chemotherapy and/or brachytherapy then surgery' *or* 'Chemotherapy then radiotherapy or concomitant radio-chemotherapy and/or brachytherapy ' at question '16 [ttt]' (In the absence of lombo-aortic involvement, what is your treatment?)

Please choose **only one** of the following:

- Yes
- No

[]You suggest performing a radical colpohysterectomy, do you associate a pelvic lymphadenectomy in the absence of suspect iliac node?

Only answer this question if the following conditions are met:
Answer was 'Surgery' *or* 'Radiotherapy or concomitant radio-chemotherapy and/or brachytherapy then surgery' *or* 'Chemotherapy then surgery ' at question '16 [ttt]' (In the absence of lombo-aortic involvement, what is your treatment?)

Please choose **only one** of the following:

- Yes
- No

Case 1 b

Same question for a 75 years old woman, without comorbidity, with a good performance status and a present entourage:

What is your current treatment for this type of cervix squamous carcinoma without lombo-aortic or pelvic involvement at the imaging?

Age (years)	75
Comorbidity	No
Autonomy	Full, lives with her husband, present entourage
FIGO	IIB
Size (mm)	50
Parametrial invasion	Bilateral proximal parametrial invasion
Adjacent organs invasion	No
Lymphovascular invasion	No
Visceral metastases	No

[]Will your treatment differ from the previous case?

Please choose **only one** of the following:

- Yes
- No

[]Do you perform a lombo-aortic surgical exploration?

Only answer this question if the following conditions are met:
Answer was 'Yes' at question '27 [chgttt2b]' (Will your treatment differ from the previous case?)

Please choose **only one** of the following:

- Yes
- No

[]In the absence of lombo-aortic involvement, what is your treatment?

Only answer this question if the following conditions are met:
Answer was 'Yes' at question '27 [chgttt2b]' (Will your treatment differ from the previous case?)

Please choose **only one** of the following:

- Surgery
- Radiotherapy or concomitant radio-chemotherapy and/or brachytherapy
- Radiotherapy or concomitant radio-chemotherapy and/or brachytherapy then surgery
- Chemotherapy then surgery
- Chemotherapy then radiotherapy or concomitant radio-chemotherapy and/or brachytherapy
- Chemotherapy alone
- Supportive care without specific cancer treatment

[]What chemotherapy regimen do you use?

Only answer this question if the following conditions are met:
Answer was 'Chemotherapy then surgery' *or* 'Chemotherapy then radiotherapy or concomitant radio-chemotherapy and/or brachytherapy ' *or* 'Chemotherapy alone' at question '29 [ttt]' (In the absence of lombo-aortic involvement, what is your treatment?)

Please choose **only one** of the following:

- Cisplatin doublet
- Carboplatin doublet
- Cisplatin alone
- Carboplatin alone
- Other combination of cytotoxics
- Other

[]Do you add Bevacizumab?

Only answer this question if the following conditions are met:
Answer was 'Chemotherapy then surgery' *or* 'Chemotherapy then radiotherapy or concomitant radio-chemotherapy and/or brachytherapy ' *or* 'Chemotherapy alone' at question '29 [ttt]' (In the absence of lombo-aortic involvement, what is your treatment?)

Please choose **only one** of the following:

- Yes
- No

[]What drug do you use in association with cisplatin?

Only answer this question if the following conditions are met:
Answer was 'Cisplatin doublet ' at question '30 [chimio]' (What chemotherapy regimen do you use?)

Please choose **only one** of the following:

- Paclitaxel
- Gemcitabine
- 5-fluorouracil
- Topotecan
- Don’t know
- Other

[]What drug do you use in association with carboplatin?

Only answer this question if the following conditions are met:
Answer was 'Carboplatin doublet' at question '30 [chimio]' (What chemotherapy regimen do you use?)

Please choose **only one** of the following:

- Paclitaxel
- Gemcitabine
- 5-fluorouracil
- Topotecan
- Don’t know
- Other

[]What drugs do you use?

Only answer this question if the following conditions are met:
Answer was 'Other combination of cytotoxics' at question '30 [chimio]' (What chemotherapy regimen do you use?)

Please write your answer(s) here:

First cytotoxic

Second cytotoxic

Third cytotoxic

[]Do you perform a pelvic external radiotherapy?

Only answer this question if the following conditions are met:
Answer was 'Chemotherapy then radiotherapy or concomitant radio-chemotherapy and/or brachytherapy ' *or* 'Radiotherapy or concomitant radio-chemotherapy and/or brachytherapy then surgery' *or* 'Radiotherapy or concomitant radio-chemotherapy and/or brachytherapy' at question '29 [ttt]' (In the absence of lombo-aortic involvement, what is your treatment?)

Please choose **only one** of the following:

- Yes
- No

[]What type of pelvic external radiotherapy do you perform?

Only answer this question if the following conditions are met:
Answer was 'Yes' at question '35 [rtexterne]' (Do you perform a pelvic external radiotherapy?)

Please choose **only one** of the following:

- Uterus, external, internal and primitive iliac nodes (up limit L4-L5): 45 Gy daily doses of 1.8-2.0 Grays
- Other regimen with adaptation of fields or physical parameters to reduce toxicity risks

[]Do you add concomitant chemotherapy to radiotherapy?

Only answer this question if the following conditions are met:
Answer was 'Yes' at question '35 [rtexterne]' (Do you perform a pelvic external radiotherapy?)

Please choose **only one** of the following:

- No
- Cisplatin alone
- Carboplatin alone
- Cisplatin+ 5-fluorouracil
- Other ____________

[]Do you perform brachytherapy?

Only answer this question if the following conditions are met:
Answer was 'Radiotherapy or concomitant radio-chemotherapy and/or brachytherapy' *or* 'Radiotherapy or concomitant radio-chemotherapy and/or brachytherapy then surgery' *or* 'Chemotherapy then radiotherapy or concomitant radio-chemotherapy and/or brachytherapy ' at question '29 [ttt]' (In the absence of lombo-aortic involvement, what is your treatment?)

Please choose **only one** of the following:

- Yes
- No

[]You suggest performing a radical colpohysterectomy, do you associate a pelvic lymphadenectomy in the absence of suspect iliac node?

Only answer this question if the following conditions are met:
Answer was 'Surgery' *or* 'Radiotherapy or concomitant radio-chemotherapy and/or brachytherapy then surgery' *or* 'Chemotherapy then surgery' at question '29 [ttt]' (In the absence of lombo-aortic involvement, what is your treatment?)

Please choose **only one** of the following:

- Yes
- No

Case 1 c

Same question for a 75 years old woman, without severe comorbidity but dependent. She is living alone, going out twice a week and needs help for shopping and cleaning:

What is your current treatment for this type of cervix squamous carcinoma without lombo-aortic or pelvic involvement at the imaging?

Age (years)	75
Comorbidity	No
Autonomy	Altered, without entourage, going out twice a week
FIGO	IIB
Size (mm)	50
Parametrial invasion	Bilateral proximal parametrial invasion
Adjacent organs invasion	No
Lymphovascular invasion	No
Visceral metastases	No

[]Will your treatment differ from the case with the younger patient?

Please choose **only one** of the following:

- Yes
- No

[]Do you perform a lombo-aortic surgical exploration?

Only answer this question if the following conditions are met:
Answer was 'Yes' at question '40 [chgttt2b]' (Will your treatment differ from the case with the younger patient?)

Please choose **only one** of the following:

- Yes
- No

[]In the absence of lombo-aortic involvement, what is your treatment?

Only answer this question if the following conditions are met:
Answer was 'Yes' at question '40 [chgttt2b]' (Will your treatment differ from the case with the younger patient?)

Please choose **only one** of the following:

- Surgery
- Radiotherapy or concomitant radio-chemotherapy and/or brachytherapy
- Radiotherapy or concomitant radio-chemotherapy and/or brachytherapy then surgery
- Chemotherapy then surgery
- Chemotherapy then radiotherapy or concomitant radio-chemotherapy and/or brachytherapy
- Chemotherapy alone
- Supportive care without specific cancer treatment

[]What chemotherapy regimen do you use?

Only answer this question if the following conditions are met:
Answer was 'Chemotherapy then surgery' *or* 'Chemotherapy then radiotherapy or concomitant radio-chemotherapy and/or brachytherapy ' *or* 'Chemotherapy alone' at question '42 [ttt]' (In the absence of lombo-aortic involvement, what is your treatment?)

Please choose **only one** of the following:

- Cisplatin doublet
- Carboplatin doublet
- Cisplatin alone
- Carboplatin alone
- Other combination of cytotoxics
- Other

[]Do you add Bevacizumab?

Only answer this question if the following conditions are met:
Answer was 'Chemotherapy then surgery' *or* 'Chemotherapy then radiotherapy or concomitant radio-chemotherapy and/or brachytherapy ' *or* 'Chemotherapy alone' at question '42 [ttt]' (In the absence of lombo-aortic involvement, what is your treatment?)

Please choose **only one** of the following:

- Yes
- No

[]What drug do you use in association with cisplatin?

Only answer this question if the following conditions are met:
Answer was 'Cisplatin doublet ' at question '43 [chimio]' (What chemotherapy regimen do you use?)

Please choose **only one** of the following:

- Paclitaxel
- Gemcitabine
- 5-fluorouracil
- Topotecan
- Don’t know
- Other

[]What drug do you use in association with carboplatin?

Only answer this question if the following conditions are met:
Answer was 'Carboplatin doublet' at question '43 [chimio]' (What chemotherapy regimen do you use?)

Please choose **only one** of the following:

- Paclitaxel
- Gemcitabine
- 5-fluorouracil
- Topotecan
- Don’t know
- Other

[]What drugs do you use?

Only answer this question if the following conditions are met:
Answer was 'Other combination of cytotoxics' at question '43 [chimio]' (What chemotherapy regimen do you use?)

Please write your answer(s) here:

First cytotoxic

Second cytotoxic

Third cytotoxic

[]Do you perform a pelvic external radiotherapy?

Only answer this question if the following conditions are met:
Answer was 'Chemotherapy then radiotherapy or concomitant radio-chemotherapy and/or brachytherapy ' *or* 'Radiotherapy or concomitant radio-chemotherapy and/or brachytherapy then surgery' *or* 'Radiotherapy or concomitant radio-chemotherapy and/or brachytherapy' at question '42 [ttt]' (In the absence of lombo-aortic involvement, what is your treatment?)

Please choose **only one** of the following:

- Yes
- No

[]What type of pelvic external radiotherapy do you perform?

Only answer this question if the following conditions are met:
Answer was 'Yes' at question '48 [rtexterne]' (Do you perform a pelvic external radiotherapy?)

Please choose **only one** of the following:

- Uterus, external, internal and primitive iliac nodes (up limit L4-L5): 45 Gy daily doses of 1.8-2.0 Grays
- Other regimen with adaptation of fields or physical parameters to reduce toxicity risks

[]Do you add concomitant chemotherapy to radiotherapy?

Only answer this question if the following conditions are met:
Answer was 'Yes' at question '48 [rtexterne]' (Do you perform a pelvic external radiotherapy?)

Please choose **only one** of the following:

- No
- Cisplatin alone
- Carboplatin alone
- Cisplatin+ 5-fluorouracil
- Other

[]Do you perform brachytherapy?

Only answer this question if the following conditions are met:
Answer was 'Radiotherapy or concomitant radio-chemotherapy and/or brachytherapy' *or* 'Radiotherapy or concomitant radio-chemotherapy and/or brachytherapy then surgery' *or* 'Chemotherapy then radiotherapy or concomitant radio-chemotherapy and/or brachytherapy ' at question '42 [ttt]' (In the absence of lombo-aortic involvement, what is your treatment?)

Please choose **only one** of the following:

- Yes
- No

[]You suggest performing a radical colpohysterectomy, do you associate a pelvic lymphadenectomy in the absence of suspect iliac node?

Only answer this question if the following conditions are met:
Answer was 'Surgery' *or* 'Radiotherapy or concomitant radio-chemotherapy and/or brachytherapy then surgery' *or* 'Chemotherapy then surgery' at question '42 [ttt]' (In the absence of lombo-aortic involvement, what is your treatment?)

Please choose **only one** of the following:

- Yes
- No

Case 2 a

What is your current treatment for this type of cervix squamous carcinoma without lombo-aortic or pelvic involvement at the imaging?

Age (years)	45
FIGO	IVA
Size (mm)	50
Parametrial invasion	Bilateral proximal parametrial invasion
Adjacent organs invasion	Rectal wall
Lymphovascular invasion	No
Visceral metastases	No

[]Do you perform a lombo-aortic surgical exploration?

Please choose **only one** of the following:

- Yes
- No

[]In the absence of lombo-aortic involvement, what is your treatment?

Please choose **only one** of the following:

- Surgery
- Radiotherapy or concomitant radio-chemotherapy and/or brachytherapy
- Radiotherapy or concomitant radio-chemotherapy and/or brachytherapy then surgery
- Chemotherapy then surgery
- Chemotherapy then radiotherapy or concomitant radio-chemotherapy and/or brachytherapy
- Chemotherapy
- Supportive care without specific cancer treatment

[]What chemotherapy regimen do you use?

Only answer this question if the following conditions are met:
Answer was 'Chemotherapy then surgery' *or* 'Chemotherapy then radiotherapy or concomitant radio-chemotherapy and/or brachytherapy ' *or* 'Chemotherapy ' at question '54 [ttt]' (In the absence of lombo-aortic involvement, what is your treatment?)

Please choose **only one** of the following:

- Cisplatin doublet
- Carboplatin doublet
- Cisplatin alone
- Carboplatin alone
- Other combination of cytotoxics
- Other

[]Do you add Bevacizumab?

Only answer this question if the following conditions are met:
Answer was 'Chemotherapy then surgery' *or* 'Chemotherapy then radiotherapy or concomitant radio-chemotherapy and/or brachytherapy ' *or* 'Chemotherapy ' at question '54 [ttt]' (In the absence of lombo-aortic involvement, what is your treatment?)

Please choose **only one** of the following:

- Yes
- No

[]What drug do you use in association with cisplatin?

Only answer this question if the following conditions are met:
Answer was 'Cisplatin doublet ' at question '55 [chimio]' (What chemotherapy regimen do you use?)

Please choose **only one** of the following:

- Paclitaxel
- Gemcitabine
- 5-fluorouracil
- Topotecan
- Don’t know
- Other

[]What drug do you use in association with carboplatin?

Only answer this question if the following conditions are met:
Answer was 'Carboplatin doublet' at question '55 [chimio]' (What chemotherapy regimen do you use?)

Please choose **only one** of the following:

- Paclitaxel
- Gemcitabine
- 5-fluorouracil
- Topotecan
- Don’t know
- Other

[]What drugs do you use?

Only answer this question if the following conditions are met:
Answer was 'Other combination of cytotoxics' at question '55 [chimio]' (What chemotherapy regimen do you use?)

Please write your answer(s) here:

First cytotoxic

Second cytotoxic

Third cytotoxic

[]Do you perform a pelvic external radiotherapy?

Only answer this question if the following conditions are met:
Answer was 'Chemotherapy then radiotherapy or concomitant radio-chemotherapy and/or brachytherapy ' *or* 'Radiotherapy or concomitant radio-chemotherapy and/or brachytherapy then surgery' *or* 'Radiotherapy or concomitant radio-chemotherapy and/or brachytherapy' at question '54 [ttt]' (In the absence of lombo-aortic involvement, what is your treatment?)

Please choose **only one** of the following:

- Yes
- No

[]What type of pelvic external radiotherapy do you perform?

Only answer this question if the following conditions are met:
Answer was 'Yes' at question '60 [rtexterne]' (Do you perform a pelvic external radiotherapy?)

Please choose **only one** of the following:

- Uterus, external, internal and primitive iliac nodes (up limit L4-L5): 45 Gy daily doses of 1.8-2.0 Grays
- Other regimen with adaptation of fields or physical parameters to reduce toxicity risks

[]Do you add concomitant chemotherapy to radiotherapy?

Only answer this question if the following conditions are met:
Answer was 'Yes' at question '60 [rtexterne]' (Do you perform a pelvic external radiotherapy?)

Please choose **only one** of the following:

- No
- Cisplatin alone
- Carboplatin alone
- Cisplatin+ 5-fluorouracil
- Other

[]Do you perform brachytherapy?

Only answer this question if the following conditions are met:
Answer was 'Radiotherapy or concomitant radio-chemotherapy and/or brachytherapy' *or* 'Radiotherapy or concomitant radio-chemotherapy and/or brachytherapy then surgery' *or* 'Chemotherapy then radiotherapy or concomitant radio-chemotherapy and/or brachytherapy ' at question '54 [ttt]' (In the absence of lombo-aortic involvement, what is your treatment?)

Please choose **only one** of the following:

- Yes
- No

[]Do you perform a pelvic lymphadenectomy?

Only answer this question if the following conditions are met:
Answer was 'Surgery' *or* 'Radiotherapy or concomitant radio-chemotherapy and/or brachytherapy then surgery' *or* 'Chemotherapy then surgery' at question '54 [ttt]' (In the absence of lombo-aortic involvement, what is your treatment?)

Please choose **only one** of the following:

- Yes
- No

Case 2 b

Same question for a 75 years old woman, without comorbidity, with a good performance status and a present entourage:

What is your current treatment for this type of cervix squamous carcinoma without lombo-aortic or pelvic involvement at the imaging?

Age (years)	75
Comorbidity	No
Autonomy	Full, lives with her husband, present entourage
FIGO	IVA
Size (mm)	50
Parametrial invasion	Bilateral proximal parametrial invasion
Adjacent organs invasion	Rectal wall
Lymphovascular invasion	No
Visceral metastases	No

[]Will your treatment differ from the previous case?

Please choose **only one** of the following:

- Yes
- No

[]Do you perform a lombo-aortic surgical exploration?

Only answer this question if the following conditions are met:
Answer was 'Yes' at question '65 [chgttt3b]' (Will your treatment differ from the previous case?)

Please choose **only one** of the following:

- Yes
- No

[]In the absence of lombo-aortic involvement, what is your treatment?

Only answer this question if the following conditions are met:
Answer was 'Yes' at question '65 [chgttt3b]' (Will your treatment differ from the previous case?)

Please choose **only one** of the following:

- Surgery
- Radiotherapy or concomitant radio-chemotherapy and/or brachytherapy
- Radiotherapy or concomitant radio-chemotherapy and/or brachytherapy then surgery
- Chemotherapy then surgery
- Chemotherapy then radiotherapy or concomitant radio-chemotherapy and/or brachytherapy
- Chemotherapy alone
- Supportive care without specific cancer treatment

[]What chemotherapy regimen do you use?

Only answer this question if the following conditions are met:
Answer was 'Chemotherapy then surgery' *or* 'Chemotherapy then radiotherapy or concomitant radio-chemotherapy and/or brachytherapy ' *or* 'Chemotherapy alone' at question '67 [ttt]' (In the absence of lombo-aortic involvement, what is your treatment?)

Please choose **only one** of the following:

- Cisplatin doublet
- Carboplatin doublet
- Cisplatin alone
- Carboplatin alone
- Other combination of cytotoxics
- Other ____________

[]Do you add Bevacizumab?

Only answer this question if the following conditions are met:
Answer was 'Chemotherapy then surgery' *or* 'Chemotherapy then radiotherapy or concomitant radio-chemotherapy and/or brachytherapy ' *or* 'Chemotherapy alone' at question '67 [ttt]' (In the absence of lombo-aortic involvement, what is your treatment?)

Please choose **only one** of the following:

- Yes
- No

[]What drug do you use in association with cisplatin?

Only answer this question if the following conditions are met:
Answer was 'Cisplatin doublet ' at question '68 [chimio]' (What chemotherapy regimen do you use?)

Please choose **only one** of the following:

- Paclitaxel
- Gemcitabine
- 5-fluorouracil
- Topotecan
- Don’t know
- Other

[]What drug do you use in association with carboplatin?

Only answer this question if the following conditions are met:
Answer was 'Carboplatin doublet' at question '68 [chimio]' (What chemotherapy regimen do you use?)

Please choose **only one** of the following:

- Paclitaxel
- Gemcitabine
- 5-fluorouracil
- Topotecan
- Don’t know
- Other

[]What drugs do you use?

Only answer this question if the following conditions are met:
Answer was 'Other combination of cytotoxics' at question '68 [chimio]' (What chemotherapy regimen do you use?)

Please write your answer(s) here:

First cytotoxic

Second cytotoxic

Third cytotoxic

[]Do you perform a pelvic external radiotherapy?

Only answer this question if the following conditions are met:
Answer was 'Chemotherapy then radiotherapy or concomitant radio-chemotherapy and/or brachytherapy ' *or* 'Radiotherapy or concomitant radio-chemotherapy and/or brachytherapy then surgery' *or* 'Radiotherapy or concomitant radio-chemotherapy and/or brachytherapy' at question '67 [ttt]' (In the absence of lombo-aortic involvement, what is your treatment?)

Please choose **only one** of the following:

- Yes
- No

[]What type of pelvic external radiotherapy do you perform?

Only answer this question if the following conditions are met:
Answer was 'Yes' at question '73 [rtexterne]' (Do you perform a pelvic external radiotherapy?)

Please choose **only one** of the following:

- Uterus, external, internal and primitive iliac nodes (up limit L4-L5): 45 Gy daily doses of 1.8-2.0 Grays
- Other regimen with adaptation of fields or physical parameters to reduce toxicity risks

[]Do you add concomitant chemotherapy to radiotherapy?

Only answer this question if the following conditions are met:
Answer was 'Yes' at question '73 [rtexterne]' (Do you perform a pelvic external radiotherapy?)

Please choose **only one** of the following:

- No
- Cisplatin alone
- Carboplatin alone
- Cisplatin+ 5-fluorouracil
- Other ____________

[]Do you perform brachytherapy?

Only answer this question if the following conditions are met:
Answer was 'Radiotherapy or concomitant radio-chemotherapy and/or brachytherapy' *or* 'Radiotherapy or concomitant radio-chemotherapy and/or brachytherapy then surgery' *or* 'Chemotherapy then radiotherapy or concomitant radio-chemotherapy and/or brachytherapy ' at question '67 [ttt]' (In the absence of lombo-aortic involvement, what is your treatment?)

Please choose **only one** of the following:

- Yes
- No

[]Do you perform a pelvic lymphadenectomy?

Only answer this question if the following conditions are met:
Answer was 'Surgery' *or* 'Radiotherapy or concomitant radio-chemotherapy and/or brachytherapy then surgery' *or* 'Chemotherapy then surgery' at question '67 [ttt]' (In the absence of lombo-aortic involvement, what is your treatment?)

Please choose **only one** of the following:

- ○ Yes
- ○ No

Case 2 c

Same question for a 75 years old woman, without severe comorbidity but dependent. She is living alone, going out twice a week and needs help for shopping and cleaning:

What is your current treatment for this type of cervix squamous carcinoma without lombo-aortic or pelvic involvement at the imaging?

Age (years)	75
Comorbidity	No
Autonomy	Altered, without entourage, going out twice a week
FIGO	IVA
Size (mm)	50
Parametrial invasion	Bilateral proximal parametrial invasion
Adjacent organs invasion	Rectal wall
Lymphovascular invasion	No
Visceral metastases	No

[]Will your treatment differ from the case with the younger patient?

Please choose **only one** of the following:

- Yes
- No

[]Do you perform a lombo-aortic surgical exploration?

Only answer this question if the following conditions are met:
Answer was 'Yes' at question '78 [chgttt3b]' (Will your treatment differ from the case with the younger patient?)

Please choose **only one** of the following:

- Yes
- No

[]In the absence of lombo-aortic involvement, what is your treatment?

Only answer this question if the following conditions are met:
Answer was 'Yes' at question '78 [chgttt3b]' (Will your treatment differ from the case with the younger patient?)

Please choose **only one** of the following:

- Surgery
- Radiotherapy or concomitant radio-chemotherapy and/or brachytherapy
- Radiotherapy or concomitant radio-chemotherapy and/or brachytherapy then surgery
- Chemotherapy then surgery
- Chemotherapy then radiotherapy or concomitant radio-chemotherapy and/or brachytherapy
- Chemotherapy alone
- Supportive care without specific cancer treatment

[]What chemotherapy regimen do you use?

Only answer this question if the following conditions are met:
Answer was 'Chemotherapy alone' *or* 'Chemotherapy then radiotherapy or concomitant radio-chemotherapy and/or brachytherapy ' *or* 'Chemotherapy then surgery' at question '80 [ttt]' (In the absence of lombo-aortic involvement, what is your treatment?)

Please choose **only one** of the following:

- Cisplatin doublet
- Carboplatin doublet
- Cisplatin alone
- Carboplatin alone
- Other combination of cytotoxics
- Other

[]Do you add Bevacizumab?

Only answer this question if the following conditions are met:
Answer was 'Chemotherapy alone' *or* 'Chemotherapy then radiotherapy or concomitant radio-chemotherapy and/or brachytherapy ' *or* 'Chemotherapy then surgery' at question '80 [ttt]' (In the absence of lombo-aortic involvement, what is your treatment?)

Please choose **only one** of the following:

- Yes
- No

[]What drug do you use in association with cisplatin?

Only answer this question if the following conditions are met:
Answer was 'Cisplatin doublet ' at question '81 [chimio]' (What chemotherapy regimen do you use?)

Please choose **only one** of the following:

- Paclitaxel
- Gemcitabine
- 5-fluorouracil
- Topotecan
- Don’t know
- Other

[]What drug do you use in association with carboplatin?

Only answer this question if the following conditions are met:
Answer was 'Carboplatin doublet' at question '81 [chimio]' (What chemotherapy regimen do you use?)

Please choose **only one** of the following:

- Paclitaxel
- Gemcitabine
- 5-fluorouracil
- Topotecan
- Don’t know
- Other

[]What drugs do you use?

Only answer this question if the following conditions are met:
Answer was 'Other combination of cytotoxics' at question '81 [chimio]' (What chemotherapy regimen do you use?)

Please write your answer(s) here:

First cytotoxic

Second cytotoxic

Third cytotoxic

[]Do you perform a pelvic external radiotherapy?

Only answer this question if the following conditions are met:
Answer was 'Radiotherapy or concomitant radio-chemotherapy and/or brachytherapy' *or* 'Radiotherapy or concomitant radio-chemotherapy and/or brachytherapy then surgery' *or* 'Chemotherapy then radiotherapy or concomitant radio-chemotherapy and/or brachytherapy ' at question '80 [ttt]' (In the absence of lombo-aortic involvement, what is your treatment?)

Please choose **only one** of the following:

- Yes
- No

[]What type of pelvic external radiotherapy do you perform?

Only answer this question if the following conditions are met:
Answer was 'Yes' at question '86 [rtexterne]' (Do you perform a pelvic external radiotherapy?)

Please choose **only one** of the following:

- Uterus, external, internal and primitive iliac nodes (up limit L4-L5): 45 Gy daily doses of 1.8-2.0 Grays
- Other regimen with adaptation of fields or physical parameters to reduce toxicity risks

[]Do you add concomitant chemotherapy to radiotherapy?

Only answer this question if the following conditions are met:
Answer was 'Yes' at question '86 [rtexterne]' (Do you perform a pelvic external radiotherapy?)

Please choose **only one** of the following:

- No
- Cisplatin alone
- Carboplatin alone
- Cisplatin+ 5-fluorouracil
- Other ____________

[]Do you perform brachytherapy?

Only answer this question if the following conditions are met:
Answer was 'Chemotherapy then radiotherapy or concomitant radio-chemotherapy and/or brachytherapy ' *or* 'Radiotherapy or concomitant radio-chemotherapy and/or brachytherapy then surgery' *or* 'Radiotherapy or concomitant radio-chemotherapy and/or brachytherapy' at question '80 [ttt]' (In the absence of lombo-aortic involvement, what is your treatment?)

Please choose **only one** of the following:

- Yes
- No

[]Do you perform a pelvic lymphadenectomy?

Only answer this question if the following conditions are met:
Answer was 'Chemotherapy then surgery' *or* 'Radiotherapy or concomitant radio-chemotherapy and/or brachytherapy then surgery' *or* 'Surgery' at question '80 [ttt]' (In the absence of lombo-aortic involvement, what is your treatment?)

Please choose **only one** of the following:

- Yes
- No

Case 3 a

What is your current treatment for this type of cervix squamous carcinoma?

Age (years)	45
FIGO	IVB
Size (mm)	50
Parametrial invasion	Bilateral proximal parametrial invasion
Adjacent organs invasion	No
Visceral metastases	Lungs and liver, asymptomatic

[]What is your treatment?

Please choose **only one** of the following:

- ○ Surgery
- ○ Radiotherapy or concomitant radio-chemotherapy and/or brachytherapy
- ○ Radiotherapy or concomitant radio-chemotherapy and/or brachytherapy then surgery
- ○ Chemotherapy then surgery
- ○ Chemotherapy then radiotherapy or concomitant radio-chemotherapy and/or brachytherapy
- ○ Chemotherapy alone
- ○ Supportive care without specific cancer treatment

[]What chemotherapy regimen do you use?

Only answer this question if the following conditions are met:
Answer was 'Chemotherapy then surgery' *or* 'Chemotherapy then radiotherapy or concomitant radio-chemotherapy and/or brachytherapy ' *or* 'Chemotherapy alone' at question '91 [ttt]' (What is your treatment?)

Please choose **only one** of the following:

- ○ Cisplatin doublet
- ○ Carboplatin doublet
- ○ Cisplatin alone
- ○ Carboplatin alone
- ○ Other combination of cytotoxics
- ○ Other ____________

[]Do you add Bevacizumab?

Only answer this question if the following conditions are met:
Answer was 'Chemotherapy then surgery' *or* 'Chemotherapy then radiotherapy or concomitant radio-chemotherapy and/or brachytherapy ' *or* 'Chemotherapy alone' at question '91 [ttt]' (What is your treatment?)

Please choose **only one** of the following:

- Yes
- No

[]What drug do you use in association with cisplatin?

Only answer this question if the following conditions are met:
Answer was 'Cisplatin doublet ' at question '92 [chimio]' (What chemotherapy regimen do you use?)

Please choose **only one** of the following:

- Paclitaxel
- Gemcitabine
- 5-fluorouracil
- Topotecan
- Don’t know
- Other ____________

[]What drug do you use in association with carboplatin?

Only answer this question if the following conditions are met:
Answer was 'Carboplatin doublet' at question '92 [chimio]' (What chemotherapy regimen do you use?)

Please choose **only one** of the following:

- Paclitaxel
- Gemcitabine
- 5-fluorouracil
- Topotecan
- Don’t know
- Other ____________

[]What drugs do you use?

Only answer this question if the following conditions are met:
Answer was 'Other combination of cytotoxics' at question '92 [chimio]' (What chemotherapy regimen do you use?)

Please write your answer(s) here:

First cytotoxic	
Second cytotoxic	
Third cytotoxic	

[]Do you perform a pelvic external radiotherapy?

Only answer this question if the following conditions are met:
Answer was 'Chemotherapy then radiotherapy or concomitant radio-chemotherapy and/or brachytherapy ' *or* 'Radiotherapy or concomitant radio-chemotherapy and/or brachytherapy then surgery' *or* 'Radiotherapy or concomitant radio-chemotherapy and/or brachytherapy' at question '91 [ttt]' (What is your treatment?)

Please choose **only one** of the following:

- Yes
- No

[]What type of pelvic external radiotherapy do you perform?

Only answer this question if the following conditions are met:
Answer was 'Yes' at question '97 [rtexterne]' (Do you perform a pelvic external radiotherapy?)

Please choose **only one** of the following:

- Uterus, external, internal and primitive iliac nodes (up limit L4-L5): 45 Gy daily doses of 1.8-2.0 Grays
- Other regimen with adaptation of fields or physical parameters to reduce toxicity risks

[]Do you add concomitant chemotherapy to radiotherapy?

Only answer this question if the following conditions are met:
Answer was 'Yes' at question '97 [rtexterne]' (Do you perform a pelvic external radiotherapy?)

Please choose **only one** of the following:

- No
- Cisplatin alone
- Carboplatin alone
- Cisplatin+ 5-fluorouracil
- Other

[]Do you perform brachytherapy?

Only answer this question if the following conditions are met:
Answer was 'Radiotherapy or concomitant radio-chemotherapy and/or brachytherapy' *or* 'Radiotherapy or concomitant radio-chemotherapy and/or brachytherapy then surgery' *or* 'Chemotherapy then radiotherapy or concomitant radio-chemotherapy and/or brachytherapy ' at question '91 [ttt]' (What is your treatment?)

Please choose **only one** of the following:

- Yes
- No

Case 3 b

Same question for a 75 years old woman, without comorbidity, with a good performance status and a present entourage:

What is your current treatment for this type of cervix squamous carcinoma?

Age (years)	75
Comorbidity	No
Autonomy	Full, lives with her husband, present entourage
FIGO	IVB
Size (mm)	50
Parametrial invasion	Bilateral proximal parametrial invasion
Adjacent organs invasion	No
Visceral metastases	Lungs and liver, asymptomatic

[]Will your treatment differ from the previous case?

Please choose **only one** of the following:

- Yes
- No

[]What is your treatment?

Only answer this question if the following conditions are met:
Answer was 'Yes' at question '101 [chgttt4b]' (Will your treatment differ from the previous case?)

Please choose **only one** of the following:

- Surgery
- Radiotherapy or concomitant radio-chemotherapy and/or brachytherapy
- Radiotherapy or concomitant radio-chemotherapy and/or brachytherapy then surgery
- Chemotherapy then surgery
- Chemotherapy then radiotherapy or concomitant radio-chemotherapy and/or brachytherapy
- Chemotherapy alone
- Supportive care without specific cancer treatment

[]What chemotherapy regimen do you use?

Only answer this question if the following conditions are met:
Answer was 'Chemotherapy then surgery' *or* 'Chemotherapy then radiotherapy or concomitant radio-chemotherapy and/or brachytherapy ' *or* 'Chemotherapy alone' at question '102 [ttt]' (What is your treatment?)

Please choose **only one** of the following:

- Cisplatin doublet
- Carboplatin doublet
- Cisplatin alone
- Carboplatin alone
- Other combination of cytotoxics
- Other

[]Do you add Bevacizumab?

Only answer this question if the following conditions are met:
Answer was 'Chemotherapy then surgery' *or* 'Chemotherapy then radiotherapy or concomitant radio-chemotherapy and/or brachytherapy ' *or* 'Chemotherapy alone' at question '102 [ttt]' (What is your treatment?)

Please choose **only one** of the following:

- Yes
- No

[]What drug do you use in association with cisplatin?

Only answer this question if the following conditions are met:
Answer was 'Cisplatin doublet ' at question '103 [chimio]' (What chemotherapy regimen do you use?)

Please choose **only one** of the following:

- Paclitaxel
- Gemcitabine
- 5-fluorouracil
- Topotecan
- Don’t know
- Other

[]What drug do you use in association with carboplatin?

Only answer this question if the following conditions are met:
Answer was 'Carboplatin doublet' at question '103 [chimio]' (What chemotherapy regimen do you use?)

Please choose **only one** of the following:

- Paclitaxel
- Gemcitabine
- 5-fluorouracil
- Topotecan
- Don’t know
- Other

[]What drugs do you use?

Only answer this question if the following conditions are met:
Answer was 'Other combination of cytotoxics' at question '103 [chimio]' (What chemotherapy regimen do you use?)

Please write your answer(s) here:

First cytotoxic

Second cytotoxic

Third cytotoxic

[]Do you perform a pelvic external radiotherapy?

Only answer this question if the following conditions are met:
Answer was 'Chemotherapy then radiotherapy or concomitant radio-chemotherapy and/or brachytherapy ' *or* 'Radiotherapy or concomitant radio-chemotherapy and/or brachytherapy then surgery' *or* 'Radiotherapy or concomitant radio-chemotherapy and/or brachytherapy' at question '102 [ttt]' (What is your treatment?)

Please choose **only one** of the following:

- Yes
- No

[]What type of pelvic external radiotherapy do you perform?

Only answer this question if the following conditions are met:
Answer was 'Yes' at question '108 [rtexterne]' (Do you perform a pelvic external radiotherapy?)

Please choose **only one** of the following:

- Uterus, external, internal and primitive iliac nodes (up limit L4-L5): 45 Gy daily doses of 1.8-2.0 Grays
- Other regimen with adaptation of fields or physical parameters to reduce toxicity risks

[]Do you add concomitant chemotherapy to radiotherapy?

Only answer this question if the following conditions are met:
Answer was 'Yes' at question '108 [rtexterne]' (Do you perform a pelvic external radiotherapy?)

Please choose **only one** of the following:

- No
- Cisplatin alone
- Carboplatin alone
- Cisplatin+ 5-fluorouracil
- Other

[]Do you perform brachytherapy?

Only answer this question if the following conditions are met:
Answer was 'Radiotherapy or concomitant radio-chemotherapy and/or brachytherapy' *or* 'Radiotherapy or concomitant radio-chemotherapy and/or brachytherapy then surgery' *or* 'Chemotherapy then radiotherapy or concomitant radio-chemotherapy and/or brachytherapy ' at question '102 [ttt]' (What is your treatment?)

Please choose **only one** of the following:

- Yes
- No

Case 3 c

Same question for a 75 years old woman, without severe comorbidity but dependent. She is living alone, going out twice a week and needs help for shopping and cleaning:

What is your current treatment for this type of cervix squamous carcinoma?

Age (years)	75
Comorbidity	No
Autonomy	Altered, without entourage, going out twice a week
FIGO	IVB
Size (mm)	50
Parametrial invasion	Bilateral proximal parametrial invasion
Adjacent organs invasion	No
Visceral metastases	Lungs and liver, asymptomatic

[]Will your treatment differ from the case with the younger patient?

Please choose **only one** of the following:

- Yes
- No

[]What is your treatment?

Only answer this question if the following conditions are met:
Answer was 'Yes' at question '112 [chgttt4b]' (Will your treatment differ from the case with the younger patient?)

Please choose **only one** of the following:

- Surgery
- Radiotherapy or concomitant radio-chemotherapy and/or brachytherapy
- Radiotherapy or concomitant radio-chemotherapy and/or brachytherapy then surgery
- Chemotherapy then surgery
- Chemotherapy then radiotherapy or concomitant radio-chemotherapy and/or brachytherapy
- Chemotherapy alone
- Supportive care without specific cancer treatment

[]What chemotherapy regimen do you use?

Only answer this question if the following conditions are met:
Answer was 'Chemotherapy alone' *or* 'Chemotherapy then radiotherapy or concomitant radio-chemotherapy and/or brachytherapy ' *or* 'Chemotherapy then surgery' at question '113 [ttt]' (What is your treatment?)

Please choose **only one** of the following:

- Cisplatin doublet
- Carboplatin doublet
- Cisplatin alone
- Carboplatin alone
- Other combination of cytotoxics
- Other

[]Do you add Bevacizumab?

Only answer this question if the following conditions are met:
Answer was 'Chemotherapy alone' *or* 'Chemotherapy then radiotherapy or concomitant radio-chemotherapy and/or brachytherapy ' *or* 'Chemotherapy then surgery' at question '113 [ttt]' (What is your treatment?)

Please choose **only one** of the following:

- Yes
- No

[]What drug do you use in association with cisplatin?

Only answer this question if the following conditions are met:
Answer was 'Cisplatin doublet ' at question '114 [chimio]' (What chemotherapy regimen do you use?)

Please choose **only one** of the following:

- Paclitaxel
- Gemcitabine
- 5-fluorouracil
- Topotecan
- Don’t know
- Other

[]What drug do you use in association with carboplatin?

Only answer this question if the following conditions are met:
Answer was 'Carboplatin doublet' at question '114 [chimio]' (What chemotherapy regimen do you use?)

Please choose **only one** of the following:

- Paclitaxel
- Gemcitabine
- 5-fluorouracil
- Topotecan
- Don’t know
- Other

[]What drugs do you use?

Only answer this question if the following conditions are met:
Answer was 'Other combination of cytotoxics' at question '114 [chimio]' (What chemotherapy regimen do you use?)

Please write your answer(s) here:

First cytotoxic

Second cytotoxic

Third cytotoxic

[]Do you perform a pelvic external radiotherapy?

Only answer this question if the following conditions are met:
Answer was 'Radiotherapy or concomitant radio-chemotherapy and/or brachytherapy' *or* 'Radiotherapy or concomitant radio-chemotherapy and/or brachytherapy then surgery' *or* 'Chemotherapy then radiotherapy or concomitant radio-chemotherapy and/or brachytherapy ' at question '113 [ttt]' (What is your treatment?)

Please choose **only one** of the following:

- Yes
- No

[]What type of pelvic external radiotherapy do you perform?

Only answer this question if the following conditions are met:
Answer was 'Yes' at question '119 [rtexterne]' (Do you perform a pelvic external radiotherapy?)

Please choose **only one** of the following:

- Uterus, external, internal and primitive iliac nodes (up limit L4-L5): 45 Gy daily doses of 1.8-2.0 Grays
- Other regimen with adaptation of fields or physical parameters to reduce toxicity risks

[]Do you add concomitant chemotherapy to radiotherapy?

Only answer this question if the following conditions are met:
Answer was 'Yes' at question '119 [rtexterne]' (Do you perform a pelvic external radiotherapy?)

Please choose **only one** of the following:

- No
- Cisplatin alone
- Carboplatin alone
- Cisplatin+ 5-fluorouracil
- Other

[]Do you perform brachytherapy?

Only answer this question if the following conditions are met:
Answer was 'Chemotherapy then radiotherapy or concomitant radio-chemotherapy and/or brachytherapy ' *or* 'Radiotherapy or concomitant radio-chemotherapy and/or brachytherapy then surgery' *or* 'Radiotherapy or concomitant radio-chemotherapy and/or brachytherapy' at question '113 [ttt]' (What is your treatment?)

Please choose **only one** of the following:

- Yes
- No

Case 4 a

What is your current treatment for this of cervix squamous carcinoma in first relapse 5 years after radio-chemotherapy?

Age (years)	45
Local relapse	No
Visceral metastases	Lungs and liver, asymptomatic

[]What is your treatment?

Please choose **only one** of the following:

- Chemotherapy
- Follow-up, then chemotherapy if the patient becomes symptomatic
- Supportive care without specific cancer treatment

[]What chemotherapy regimen do you use?

Only answer this question if the following conditions are met:
Answer was 'Follow-up, then chemotherapy if the patient becomes symptomatic' *or* 'Chemotherapy ' at question '123 [ttt]' (What is your treatment?)

Please choose **only one** of the following:

- Cisplatin doublet
- Carboplatin doublet
- Cisplatin alone
- Carboplatin alone
- Other combination of cytotoxics
- Other

[]Do you add Bevacizumab?

Only answer this question if the following conditions are met:
Answer was 'Follow-up, then chemotherapy if the patient becomes symptomatic' *or* 'Chemotherapy ' at question '123 [ttt]' (What is your treatment?)

Please choose **only one** of the following:

- Yes
- No

[]What drug do you use in association with cisplatin?

Only answer this question if the following conditions are met:
Answer was 'Cisplatin doublet ' at question '124 [chimio]' (What chemotherapy regimen do you use?)

Please choose **only one** of the following:

- Paclitaxel
- Gemcitabine
- 5-fluorouracil
- Topotecan
- Don’t know
- Other

[]What drug do you use in association with carboplatin?

Only answer this question if the following conditions are met:
Answer was 'Carboplatin doublet' at question '124 [chimio]' (What chemotherapy regimen do you use?)

Please choose **only one** of the following:

- Paclitaxel
- Gemcitabine
- 5-fluorouracil
- Topotecan
- Don’t know
- Other

[]What drugs do you use?

Only answer this question if the following conditions are met:
Answer was 'Other combination of cytotoxics' at question '124 [chimio]' (What chemotherapy regimen do you use?)

Please write your answer(s) here:

First cytotoxic

Second cytotoxic

Third cytotoxic

Case 4 b

Same question for a 75 years old woman, without comorbidity, with a good performance status and a present entourage:

What is your current treatment for this of cervix squamous carcinoma in first relapse 5 years after radio-chemotherapy?

Age (years)	75
Comorbidity	No
Autonomy	Full, lives with her husband, present entourage
Local relapse	No
Visceral metastases	Lungs and liver, asymptomatic

[]Will your treatment differ from the previous case?

Please choose **only one** of the following:

- Yes
- No

[]What is your treatment?

Only answer this question if the following conditions are met:
Answer was 'Yes' at question '129 [chgttt5b]' (Will your treatment differ from the previous case?)

Please choose **only one** of the following:

- Chemotherapy
- Follow-up, then chemotherapy if the patient becomes symptomatic
- Supportive care without specific cancer treatment

[]What chemotherapy regimen do you use?

Only answer this question if the following conditions are met:
Answer was 'Follow-up, then chemotherapy if the patient becomes symptomatic' *or* 'Chemotherapy ' at question '130 [ttt]' (What is your treatment?)

Please choose **only one** of the following:

- Cisplatin doublet
- Carboplatin doublet
- Cisplatin alone
- Carboplatin alone
- Other combination of cytotoxics
- Other

[]Do you add Bevacizumab?

Only answer this question if the following conditions are met:
Answer was 'Follow-up, then chemotherapy if the patient becomes symptomatic' *or* 'Chemotherapy ' at question '130 [ttt]' (What is your treatment?)

Please choose **only one** of the following:

- ◯ Yes
- ◯ No

[]What drug do you use in association with cisplatin?

Only answer this question if the following conditions are met:
Answer was 'Cisplatin doublet ' at question '131 [chimio]' (What chemotherapy regimen do you use?)

Please choose **only one** of the following:

- ◯ Paclitaxel
- ◯ Gemcitabine
- ◯ 5-fluorouracil
- ◯ Topotecan
- ◯ Don’t know
- ◯ Other ________

[]What drug do you use in association with carboplatin?

Only answer this question if the following conditions are met:
Answer was 'Carboplatin doublet' at question '131 [chimio]' (What chemotherapy regimen do you use?)

Please choose **only one** of the following:

- ◯ Paclitaxel
- ◯ Gemcitabine
- ◯ 5-fluorouracil
- ◯ Topotecan
- ◯ Don’t know
- ◯ Other ________

[]What drugs do you use?

Only answer this question if the following conditions are met:
Answer was 'Other combination of cytotoxics' at question '131 [chimio]' (What chemotherapy regimen do you use?)

Please write your answer(s) here:

First cytotoxic	
Second cytotoxic	
Third cytotoxic	

Case 4 c

Same question for a 75 years old woman, without severe comorbidity but dependent. She is living alone, going out twice a week and needs help for shopping and cleaning:

What is your current treatment for this cervix squamous carcinoma in first relapse 5 years after radio-chemotherapy?

Age (years)	75
Comorbidity	No
Autonomy	Altered, without entourage, going out twice a week
Local relapse	No
Visceral metastases	Lungs and liver, asymptomatic

[]Will your treatment differ from the case with the younger patient?

Please choose **only one** of the following:

- Yes
- No

[]What is your treatment?

Only answer this question if the following conditions are met:
Answer was 'Yes' at question '136 [chgttt5b]' (Will your treatment differ from the case with the younger patient?)

Please choose **only one** of the following:

- Chemotherapy
- Follow-up, then chemotherapy if the patient becomes symptomatic
- Supportive care without specific cancer treatment

[]What chemotherapy regimen do you use?

Only answer this question if the following conditions are met:
Answer was 'Chemotherapy ' *or* 'Follow-up, then chemotherapy if the patient becomes symptomatic' at question '137 [ttt]' (What is your treatment?)

Please choose **only one** of the following:

- Cisplatin doublet
- Carboplatin doublet
- Cisplatin alone
- Carboplatin alone
- Other combination of cytotoxics
- Other

[]Do you add Bevacizumab?

Only answer this question if the following conditions are met:
Answer was 'Chemotherapy ' *or* 'Follow-up, then chemotherapy if the patient becomes symptomatic' at question '137 [ttt]' (What is your treatment?)

Please choose **only one** of the following:

- Yes
- No

[]What drug do you use in association with cisplatin?

Only answer this question if the following conditions are met:
Answer was 'Cisplatin doublet ' at question '138 [chimio]' (What chemotherapy regimen do you use?)

Please choose **only one** of the following:

- Paclitaxel
- Gemcitabine
- 5-fluorouracil
- Topotecan
- Don’t know
- Other

[]What drug do you use in association with carboplatin?

Only answer this question if the following conditions are met:
Answer was 'Carboplatin doublet' at question '138 [chimio]' (What chemotherapy regimen do you use?)

Please choose **only one** of the following:

- Paclitaxel
- Gemcitabine
- 5-fluorouracil
- Topotecan
- Don’t know
- Other

[]What drugs do you use?

Only answer this question if the following conditions are met:
Answer was 'Other combination of cytotoxics' at question '138 [chimio]' (What chemotherapy regimen do you use?)

Please write your answer(s) here:

First cytotoxic	
Second cytotoxic	
Third cytotoxic	

Thank you for taking our survey.

The next step of this project will be a prospective study of the patients of more than 65 years treated for a cervix cancer.

If you wish to be kept informed of the results of this survey or to have more informations about the next study, send an e-mail at elderly.cc@gmail.com

Submit your survey.
Thank you for completing this survey.
